# Leptin differentially regulate STAT3 activation in *ob/ob* mouse adipose mesenchymal stem cells

**DOI:** 10.1186/1743-7075-9-109

**Published:** 2012-12-05

**Authors:** Zhou Zhou, Hui Ren Zhou, Dayong Wu, Chia-Cheng Chang, Naima Moustaid-Moussa, Kate J Claycombe

**Affiliations:** 1 Department of Food Science and Human Nutrition, Michigan State University, East Lansing, Michigan, MI 48824, USA; 2 Comparative Medicine and Integrative Biology Graduate Program, College of Veterinary Medicine, Michigan State University, East Lansing, MI, 48824, USA; 3 Nutritional Immunology Laboratory, JM USDA HNRC at Tufts University, Boston, MA, 02111, USA; 4 Department of Pediatrics and Human Development, Michigan State University, East Lansing, MI, 48824, USA; 5 Department of Animal Science, and University of Tennessee Obesity Research Cente, University of Tennessee, Knoxville, TN, 37996, USA; 6 Current affiliation: Dana-Farber Cancer Institute, Harvard Medical School, Boston, MA, 02135, USA; 7 USDA-ARS, Grand Forks Human Nutrition Research Center, 2420 2nd Ave. N., Stop 9034, Grand Forks, ND 58203, USA

**Keywords:** Obesity, Adipose stem cell, Leptin

## Abstract

**Background:**

Leptin-deficient *ob/ob* mice exhibit adipocyte hypertrophy and hyperplasia as well as elevated adipose tissue and systemic inflammation. Multipotent stem cells isolated from adult adipose tissue can differentiate into adipocytes *ex vivo* and thereby contribute toward increased adipocyte cell numbers, obesity, and inflamm ation. Currently, information is lacking regarding regulation of adipose stem cell numbers as well as leptin-induced inflammation and its signaling pathway in *ob/ob* mice.

**Methods:**

Using leptin deficient *ob/ob* mice, we investigated whether leptin injection into *ob/ob* mice increases adipose stem cell numbers and adipose tissue inflammatory marker MCP-1 mRNA and secretion levels. We also determined leptin mediated signaling pathways in the adipose stem cells.

**Results:**

We report here that adipose stem cell number is significantly increased following leptin injection in *ob/ob* mice and with treatment of isolated stem cells with leptin *in vitro*. Leptin also up-regulated MCP-1 secretion in a dose- and time-dependent manner. We further showed that increased MCP-1 mRNA levels were due to increased phosphorylation of Signal Transducer and Activator of Transcription 3 (STAT3) Ser727 but not STAT3 Tyr705 phosphorylation, suggesting differential regulation of MCP-1 gene expression under basal and leptin-stimulated conditions in adipose stem cells.

**Conclusions:**

Taken together, these studies demonstrate that leptin increases adipose stem cell number and differentially activates STAT3 protein resulting in up-regulation of MCP-1 gene expression. Further studies of mechanisms mediating adipose stem cell hyperplasia and leptin signaling in obesity are warranted and may help identify novel anti-obesity target strategies.

## Background

Obesity increases the risk for developing Type 2 Diabetes (T2D), cardiovascular disease (CVD), and cancer [1]. The elevated risk has been suggested to be mediated, in part, by increased chronic inflammation [2]. Elevated secretion of pro-inflammatory cytokines with obesity is significantly reduced with weight loss, particularly with decreased body fat [3,4]. These findings suggest that adipose tissue is an important source of obesity-associated inflammation. We previously showed that adipose tissue stem cells (CD34^+^) isolated from leptin-deficient obese *ob/ob* mice secrete high levels of Monocyte Chemotactic Protein-1 (MCP-1) [5]. This adipokine may have a more profound effect on adipose tissue inflammation, compared to other inflammatory factors secreted from adipose tissue, such as tumor necrosis factor-alpha (TNF-alpha), interleukin(IL)-1, IL-6, IL-8, and IL-18 [6]. This may be due to MCP-1’s chemotatic activity which causes infiltration and accumulation of macrophages (Mϕ) in adipose tissue [7-9], thus further exacerbating adipose tissue inflammation [7-9].

Leptin is considered to be a member of the pro-inflammatory IL-6 family of cytokines [10]. Leptin modulates humoral and cell-mediated immune responses [11-15]. In agreement with this immune regulatory role, the long form of leptin receptor (Ob-R_L_) is expressed in immune cells such as monocytes and T cells [16], dendritic cells [17], eosinophils [18], B cells [19], and Mϕ [20]. Leptin receptors are localized on adipose tissue cells [21]. Leptin binding to the leptin receptor stimulates stem cell proliferation [20], differentiation [22,23] and cytokine secretion from adipose tissue [24], possibly via Janus Kinase (JAK)-Signal Transducer and Activator of Transcription 3 (STAT3) signaling pathways [25]. Typically, leptin induces proinflammatory cytokine expression also via activation of leptin receptor followed by activation of JAK 2/3 and STAT3 pathways [12,26]. In addition to JAK2/3-STAT3 pathway, other non-traditional cytokine type receptor signaling molecules such as insulin receptor substrate-1 (IRS-1), phosphoinositide 3-kinase (PI3K), protein kinase B/Akt (PKB/Akt) also mediate the proinflammatory effects of leptin [27,28]. Interestingly, studies have shown that MCP-1 gene expression is regulated in STAT3 dependent manner [29]. Previously, we have shown that differences in serum MCP-1 between lean and leptin deficient obese (*ob/ob*) mice might be due to an increased MCP-1 expression in adipose tissue; in addition, among the adipose tissue subtype cells that are capable of secreting MCP-1, CD34^+^ cells are the primary contributors toward elevated serum MCP-1 levels in the *ob/ob* mice [5]. It is plausible that CD34^+^ cells play key role in obesity-associated inflammation since obesity is associated with elevated levels of inflammation and that CD34^+^ cells contribute to the elevated inflammation by secreting MCP-1. Recently, a subpopulation of mouse CD34^+^ cells has been identified as precursors of white adipocytes, bone, cartilage, and muscle *in vitro*[30]. Additional studies have demonstrated that cell surface markers such as CD34 and stem cell antigen 1 (Sca-1) are found in adipose-derived mesenchymal stem cells (Ad-MSCs) [30]. Therefore, we hypothesized that MCP-1 secreting CD34^+^ cells from *ob/ob* mice adipose tissue have mesenchymal stem cell (MSC) phenotypes. In addition, several obesity-associated factors and conditions such as hyperglycemia, hyperinsulinemia, and inflammation, all of which are present in *ob/ob* mice [31-34], can induce MCP-1 secretion from adipocytes [35-37]. Therefore, it is plausible that a common adipose-derived and circulating factor such as leptin, accounts for increased MCP-1 as well as the above metabolic alterations in obesity. Leptin-mediated MCP-1 secretion has been shown in immune cells such as eosinophils [38]. However, no studies addressed whether leptin injection into leptin deficient ob/ob mice increases plasma MCP-1 concentration.

The current study tested whether adipose tissue mass, numbers of adipose tissue SVF cells and MSC numbers are altered with obesity in the *ob/ob* model. The current study also tested whether leptin injection to *ob/ob* mice increased plasma and adipose MCP-1expression and further dissected the intracellular signaling pathways involved in adipose MCP-1expression.

## Methods

### Mice

Four-month-old male wild-type C57BL/6J and leptin-deficient obese C57BL/6J-*ob/ob (ob/ob)* mice were purchased from The Jackson Laboratory (Bar Harbor, ME). Epididymal white adipose tissue samples were used to isolate adipose tissue cells. The study protocol was reviewed and approved by the Michigan State University Institutional Animal Care and Use Committee. 

**Cell culture Reagents.** Recombinant murine leptin was purchased from PeproTech (Rocky Hill, NJ) with an endotoxin level less than 0.1 ng per μg (1EU/μg). Inhibitors: JAK inhibitor I, AG 490, Akt inhibitor IV and U-0126 were purchased from Calbiochem (La Jolla, CA). LY294002 was purchased from Cell Signaling Technology, Inc. (Danvers, MA). Rabbit polyclonal antibodies against phospho-STAT3 (Tyr705), phospho-STAT3 (Ser727), STAT3, phospho-p44/42 MAP kinase (Thr202/Tyr204), p44/42 MAP kinase, phospho-Akt (Ser473), phospho-Akt (Thr308), Akt (pan) rabbit mAb, phospho-PI3K p85(Tyr458) and PI3 Kinase p85 rabbit mAb were purchased from Cell Signaling Technology. 

**Isolation of stromal vascular fraction (SVF) and magnetic cell sorting (MACS) analysis.** Epididymal white adipose tissue was excised from lean control and *ob/ob* mice. Samples were minced and digested using 0.25% collagenase (2 mg/ml of collagenase type I, Worthington Biochemical, Lakewood, NJ) in Hanks' balanced salt solution (HBSS) at 37°C and digested adipose tissue cells were filtered through 100-μm nylon cell strainers (BD Biosciences, Bedford, MA). The floating adipocytes were removed after centrifugation (450 X g), and the pellet was washed and resuspended in DMEM supplemented with 10% heat-inactivated fetal bovine serum (HIFBS; GIBCO, Grand Island, New York), 100 IU penicillin (P), and 100 μg/ml streptomycin (S). The resulting SVF cell pellet was treated with red blood cell lysis buffer, washed with PBS and resuspended in DMEM for hemocytometer counting and isolation of CD34^+^ cells. SVF cells were first stained with 10 μg each of R-phycoerythrin (PE)-conjugated anti-CD34 (ebioscience, San Diego, CA), Sca-1 (eBioscience), CD45 (eBioscience), and F4/80 antibodies (Invitrogen, Carlsbad, CA) by mixing and incubating these antibodies separately with 1×10^7^ cells in 100 μl of MACS buffer (Miltenyi Biotec, Auburn, CA) with 10 μl of blocking buffer (Miltenyi Biotec). The cells were then incubated for 10 min in the dark at 4°C, washed, and magnetically labeled with 20 μl anti-PE microbeads (Miltney Biotec), by resuspending cells with PE microbeads in 80 μl of MACS buffer for 15 min at 4°C. The cells were washed and resuspended in 1 ml of MACS buffer, and loaded on a magnetic column that was placed in MACS separator in order to retain the magnetically labeled cells and to elute unlabeled cells. After removal of the column from the magnetic separator, the CD34^+^ cells were eluted with MACS buffer and resuspended in DMEM medium with 10% HIFBS and P/S. Cells were then plated in 60 mm cell culture dishes, grown to confluence, and subcultured or cryopreserved for further studies. 

**Fluorescence activated cell sorting (FACS) analysis.** Mouse CD34, Sca-1, and CD45 FITC or PE conjugated antibodies and their IgG isotype control antibodies were purchased from BD Biosciences. MACS-separated CD34^+^ cells were resuspended in 500 μl of fixation buffer (eBioscience) followed by room temperature incubation for 15 min. The resulting cells were washed with the staining buffer (eBioscience) twice, centrifuged at 500 rpm for 5 min at 4°C and resuspended in 300 μl staining buffer containing appropriate antibody at 1 μg/1x10^6^ cells. Antibody solution containing cells was then incubated in the dark for 1 hr at room temperature, washed in staining buffer twice and then resuspended in 500 μl of staining buffer for FACS analysis. The stained samples were analyzed by FACS Vantage equipped with a G3 Mac computer and CellQuest software (Becton-Dickinson, San Jose, CA). Single- and two-color controls were used to set the lower limit of positive fluorescence and compensation for spectral overlap of these fluorochromes. Data based on 20,000–50,000 events were acquired from each sample and analyzed using WinLis 5.0 (Verity Software House, Topsham, ME). 

**Differentiation of Sca-1**^**+**^**/CD45**^**-**^**/CD34**^**+**^**cells.** MACS-separated early passage (passage 3) cells as well as cells expanded in different culture conditions for various time periods were used for differentiation studies. The cells were treated with different induction cocktails in D medium (a modified Eagle’s Minimum Essential Medium) with 10% FBS. All studies were carried out with same number of controls. For osteogenesis, cells were plated at the seeding density of 3,000 cells/cm^2^ in 6-well plates (regular or laminin-coated) and treated with dexamethasone (0.1 μM), L-ascorbic acid 2-phosphate (Asc 2P, 50 μM) and β-glycerophosphate disodium (10 mM) (DAG cocktail) in D medium containing 10% FBS for 4 weeks, with medium change once in every 3 days. We optimized the differentiation protocol by omitting dexamethasone from the induction regimen, and treating the cells with 5-fold higher concentration of Asc 2P (250 μM), and β-glycerophosphate disodium (10 mM) (AG cocktail) in D medium containing 10% FBS for 4 weeks, with medium changed once every 3 days. Alizarin red staining was performed to detect calcified extracellular matrix deposits. For chondrogenesis, the micromass culture method was used. 1 × 10^5^ cells in 10 μL volume were plated in each of 3 wells in a 24-well plate and incubated for 2.5 hours and then treated by transforming growth factor-beta 1 (TGF-β1,10 ng/ml), Asc 2P (50 μM) and insulin (6.25 μg/ml) (TAI cocktail) for 14 days, with medium change once every 3 days. The micromasses were stained with Alcian blue to detect the presence of sulfated proteoglycan-rich matrix. For adipogenesis, cells were plated at the seeding density of 10,000 cells/cm^2^ in 6-well plates, and treated with the standard protocol of 3-isobutyl-1-methylxanthine (IBMX, 500 μM), dexamethasone (1 μM), indomethacin (100 μM), and insulin (10 μg/ml) (IDII cocktail) for 21 days, with medium change once in every 3 days. We optimized the adipogenic induction cocktail for these CD34^+^ mAd-MSCs as follows. Cells were plated at the seeding density of 5000 cells/cm^2^ in 60 mm plates, and treated with prostaglandin J2 (15 μM) (Cayman Chemical), dexamethasone (1.5 μM), insulin (600 nM), and glucose (6.75 mg/ml) (PDIG cocktail) for 14 days, with medium change once in every 3 days. Oil Red O staining was done to examine the lipid droplet formation. 

**Enzyme-Linked Immunosorbant Assay (ELISA).** MCP-1 ELISA was performed according to the manufacturer’s procedure using a mouse CCL2/JE Duoset ELISA Development Kit (R & D systems, Minneapolis, MN). Briefly, MCP-1 antibodies were immobilized in microtiter plates. MCP-1 standard solutions and the supernatant from different treatment were applied to the pre-coated wells and incubated for 2h at RT. Unbound proteins were washed away and MCP-1-antibody was added to each well and incubated for 2h at RT. Next, HRP-conjugated MCP-1-antibody was added to each well and incubated for 20min at RT after washing. HRP substrate was added and further incubated at RT for 20 min following washing steps. The reaction was stopped and absorbance was measured in the microtiter plate reader (Molecular Devices Corporation, Menlo Park, CA) at 450 nm and 570 nm for λ-correction.

**Real Time PCR.** Total RNA was extracted from Sca-1^+^/CD45^-^/CD34^+^ cells using a RNeasy Mini Kit (Qiagen, Valencia, CA). 100 ng/μl of total RNA were used to measure MCP-1 mRNA by real time PCR. The primers, probe and endogenous control (18S-rRNA) were purchased as Taqman assay reagents (Applied Biosystems, Foster City, CA). Taqman One Step PCR Master Mix (Applied Biosystems) was used to quantify MCP-1 and 18S-rRNA following manufacturer’s instructions on an ABI Prism 7900 (Applied Biosystems). 18S-rRNA was used to normalize target gene expression. Target gene expression levels were calculated relative to the control group. For PCR assays, total RNAs were extracted from cells using Versagene RNA Purification Kit (Gentra) and treated with DNase I (Turbo DNA-free) (Ambion) to remove contaminating DNA. cDNAs were synthesized from 1 μg total RNA using anchored oligo dT primers and Superscript III reverse transcriptase (Invitrogen). Primers derived from coding regions of respective genes in mouse genome were used to amplify the target sites. To ensure that the primers would uniquely amplify the target transcripts, primers for some of the genes (Osteocalcin, Fabp4, Lpl) were designed to flank an intron, which allows to further rule out genomic contamination by simple inspection of product size. PCR reactions were prepared with 2μl cDNA, 5 pmol of each primer, 0.5 units of Taq polymerase (Invitrogen, CA), and final concentrations of 40 μM dNTPs, 2 mM MgCl2, 20 mM Tris–HCl, and 50 μl KCl. Cycling conditions were as follows: 94°C for 4 min; 30 cycles at 94°C for 1 min, 60°C for 1 min, 72°C for 1 min; followed by 72°C for 5 min. The PCR products were separated on 2% agarose gel by electrophoresis, stained with ethidium bromide, visualized under UV light, and digital images captured with AlphaImager software. Respective tissue samples (bone, cartilage, and fat from mouse) were used as positive controls to validate the primers, Gapdh was used as a housekeeping gene, and no template controls (water instead of cDNA) were used as negative controls. 

**Western blot analysis.** Cells were washed with ice-cold PBS and then lysed in boiling lysis buffer (1% [w/v], Sodium dodecylsulfate, 1mM sodium ortho-vanadate and 10 mM Tris (pH 7.4) for 5 min followed by brief sonication. The lysate was centrifuged at 12,000 x g for 15 min at 4°C. Protein concentrations were measured with a Bio-Rad DC protein assay kit (Bio Rad Laboratories Inc., Melville, NY). Total cellular proteins were resolved by 7.5% (w/v) acrylamide gel and transferred to a polyvinylidene difluoride (PVDF) membrane (Amersham, Arlington Heights, IL). The membrane was blocked with 5% non-fat milk in Tris-buffered saline (TBS) containing 0.1% Tween 20 for 1 hour at room temperature. The membrane was incubated overnight at 4°C with the one of the following specific primary antibodies (diluted with 5% bovine serum albumin in TBS containing 0.1% Tween 20), followed by HRP-conjugated anti-rabbit IgG antibodies (Amersham). Bound peroxidase was determined using an ECL Chemiluminescence detection Kit (Amersham). Western analysis was conducted using primary antibodies specific for Phospho-STAT3 (Tyr705) (1:1000), Phospho-STAT3 (Ser727) (1:1000), STAT3 (1:1000), Phospho-p44/42 MAP kinase (Thr202/Tyr204) (1:1000), p44/42 MAP kinase (1:1000), Phospho-Akt (Ser473) (1:1000), Phospho-Akt (Thr308) (1:1000), Akt (pan) rabbit mAb (1:1000), Phospho-PI3K p85(Tyr458) (1:1000) and PI3 Kinase p85 rabbit mAb (1:1000). To assess loading, membranes were stripped and reprobed with specific antibodies that recognize both phosphorylated and unphosphorylated forms of each protein.

**Figure 1:**
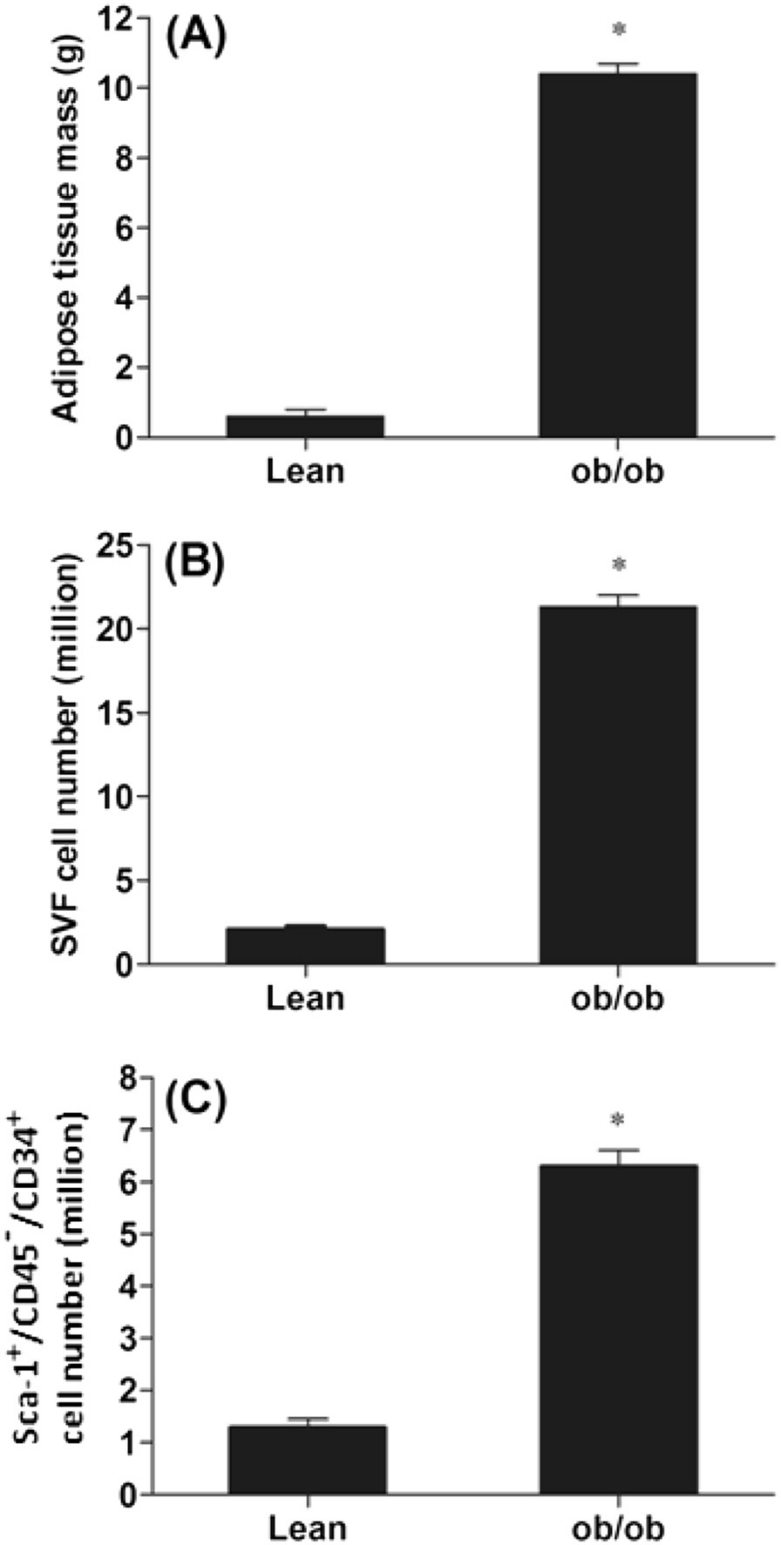
**Leptin increases plasma MCP-1 and adipose tissue MCP-1 mRNA.** (**A**). Epididymal adipose tissue MCP-1 mRNA of lean and *ob/ob* mice injected with leptin (1 μg/g body weight) for 3 hrs. (**B**). Plasma MCP-1 concentration of lean and *ob/ob* mice injected with leptin (1 μg/g body weight) for 3 hours. For (**A**)-(**B**), the mean ± SE for eight mice per group are plotted, ** represents *p*<0.001compared to lean mice and ^##^ represents *p*<0.001compared to *ob/ob* mice.

### Statistical analyses

Data were reported as mean ± standard error of the mean (SEM) and were analyzed by the general linear model (GLM) ANOVA and pairwise comparisons made by Bonferroni method by using Sigma Stat software (Jandel Scientific, San Rafael, CA) when appropriate. Means with different letters differ at *p* < 0.05.

## Results

### Obesity-associated increase in SVF and Sca-1^+^/CD45^-^/CD34^+^ cell numbers

Using age-matched littermate lean and *ob/ob* mice, we isolated adipose tissue SVF cells as well as Sca-1^+^/CD45^-^/CD34^+^ MSCs. As shown in Figure 1A, adipose tissue mass was significantly greater (>10×) in the *ob/ob* mice compared to lean mice. Results also showed that SVF cell numbers of *ob/ob* mice were significantly greater than that of lean mice (Figure 1B; 22.7 ±2.41 × 10^6^ in *ob/ob* versus 2.49 ± 0.14 × 10^6^ in lean mice; *p*<0.001). Similarly, Sca-1^+^/CD45^-^/CD34^+^ cell numbers were greater in obese versus lean animals (Figure 1C; 6.3 ±1.27 × 10^6^ in *ob/ob* versus 1.4 ± 0.1 × 10^6^ in lean mice; *p*<0.05).

**Figure 2:**
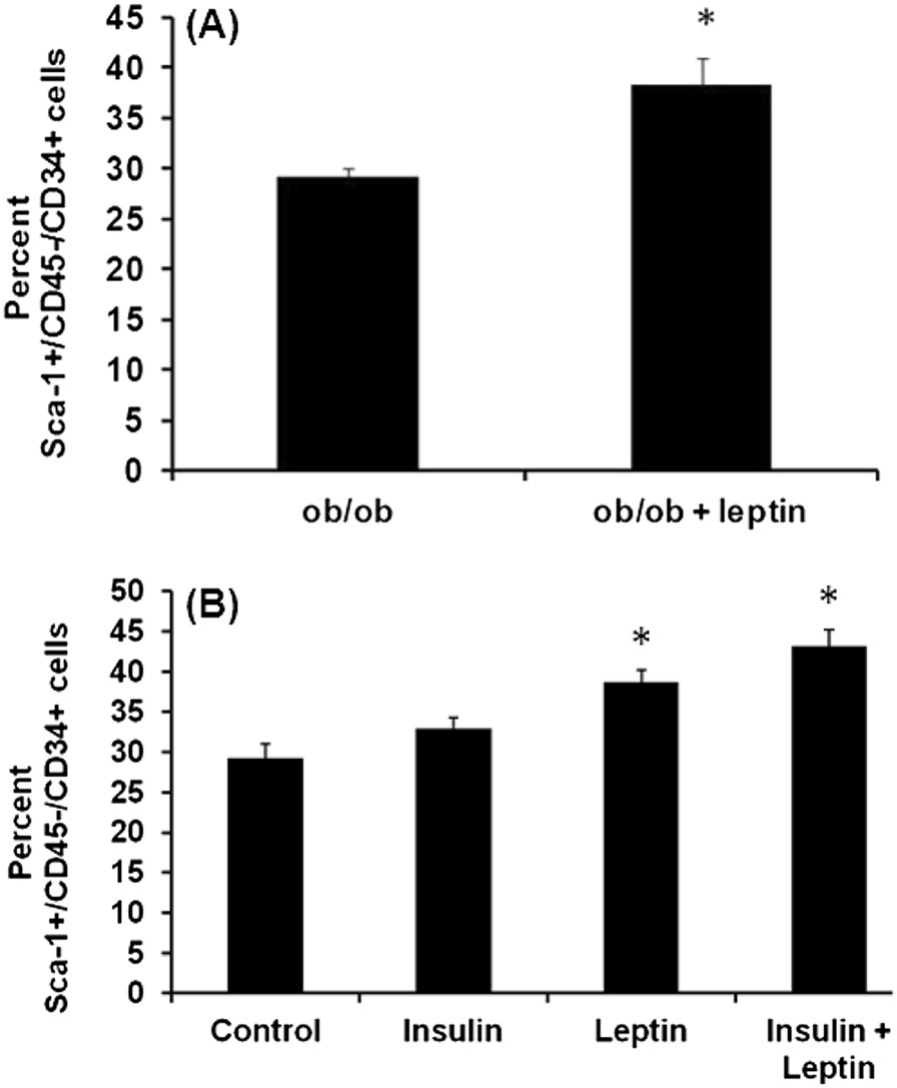
**Adipose tissue mass, SVF cell and Sca-1**^**+**^**/CD45**^**-**^**/CD34**^**+**^**cell numbers are increased in *ob/ob *mice.** (**A**). Adipose tissue mass of C57BL/6J (lean) and *ob*/*ob* (leptin-deficient) mice. (**B**). Stromal vascular fraction (SVF) cells were isolated from epididymal white adipose tissue from lean and *ob/ob* mice and SVF cell numbers were quantified using FACS analysis. (**C**). CD34^+^ cells isolated using magnetic column were stained for Sca-1 and CD45 and quantified using FACS analysis. For (**A**)-(**C**), the mean ± SE for eight mice per group are plotted, *p<0.05 compared to lean mice.

Data demonstrated that the total numbers of SVF cells and CD34^+^/Sca-1^+^/CD45^-^ cells were increased in the adipose tissue in *ob/ob* mice coincident with an equal increase in total fat mass. In contrast to expectation, normalization of SVF cell numbers by adipose tissue mass showed decreases in SVF cell numbers per gram of adipose tissue in *ob/ob* mice. In addition, the percent of CD34^+^/Sca-1^+^/CD45^-^ cells in SVF were also reduced in *ob/ob* mice because of the increased SVF cell numbers in *ob/ob* mice.

### Leptin increases Sca-1^+^/CD45^-^/CD34^+^ cell numbers *in vivo* and *in vitro*

To test the effects of leptin on Sca-1^+^/CD45^-^/CD34^+^ cell numbers *in vivo*, SVF cells from the *ob/ob* mice that were injected with saline or leptin (1 μg/gm body weight) for 10 days were isolated. The SVF cells were then analyzed for Sca-1^+^/CD45^-^/CD34^+^ cell numbers. Data showed that leptin injection increases percent Sca-1^+^/CD45^-^/CD34^+^ cell numbers (Figure 2A, *p*<0.05). To test the effects of leptin *in vitro*, isolated Sca-1^+^/CD45^-^/CD34^+^ cells were treated with insulin (1000 ng/ml), leptin (1000 ng/ml) and leptin (1000 ng/ml) + insulin (1000 ng/ml) for 96 h. Data showed that leptin and leptin + insulin increase Sca-1^+^/CD45^-^/CD34^+^ cell numbers (Figure 2B, *p*<0.05).

**Figure 3:**
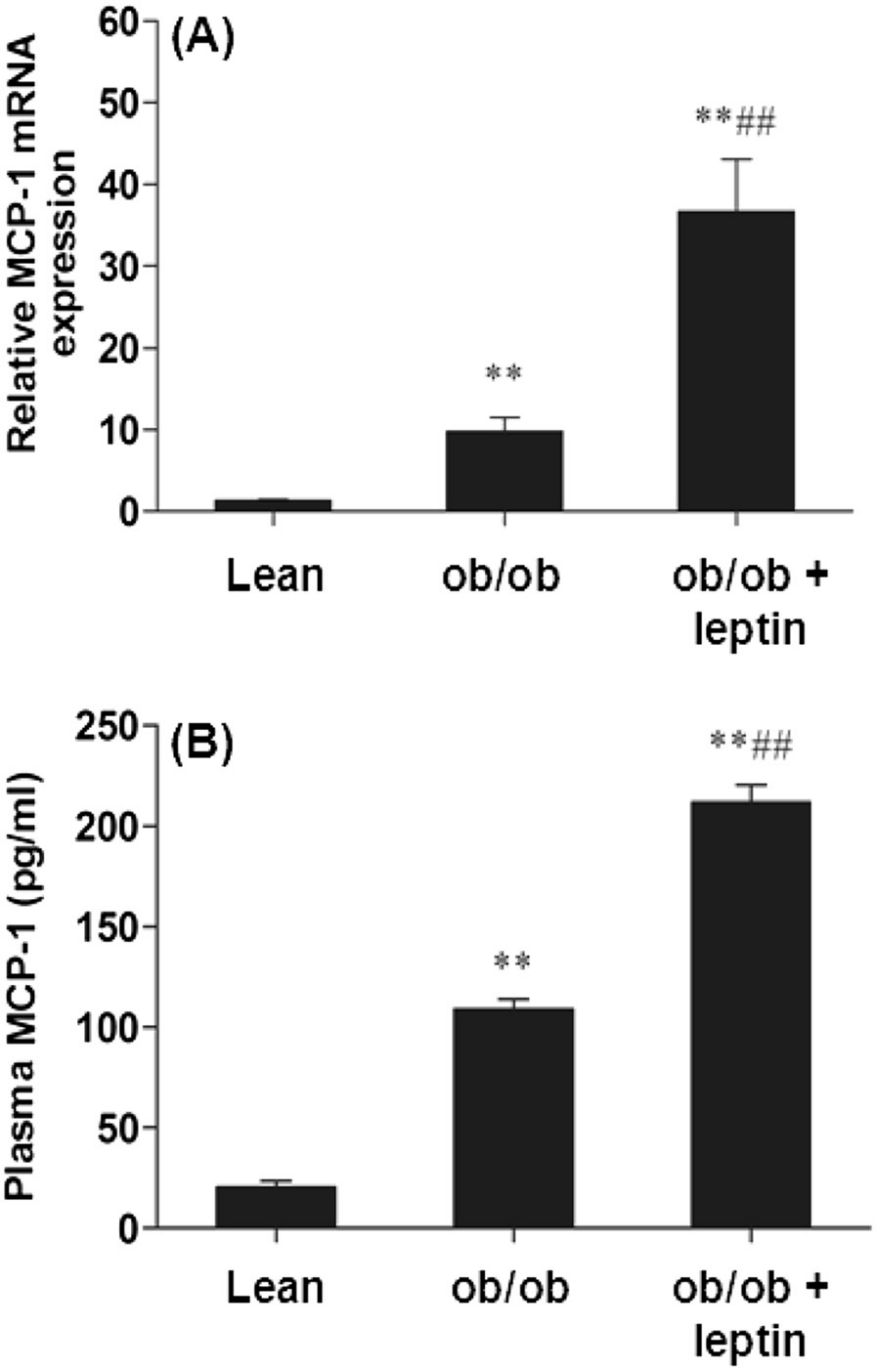
**Leptin increases Sca-1**^**+**^**/CD45**^**-**^**/CD34**^**+**^**cell numbers *in vivo *and *in vitro*.** (**A**). Cell numbers were measured using FACS analysis of SVF cells that were isolated from leptin-injected *ob/ob* mice (1 μg/g body weight for 10 days). The mean ± SE for three mice per group are plotted, *p<0.05 compared to lean mice (**B**). Isolated and cultured **SVF** cells from ob/ob mice were treated with insulin (1000 ng/ml), leptin (1000 ng/ml) and leptin (1000 ng/ml) + insulin (1000 ng/ml) for 96h. Cells were quantified using FACS analysis. Data are presented as mean ± SE, triplicate samples of *n* = 3 independent experiments, * represents *p*<0.05.

### Effects of leptin injection to *ob/ob* mice on Sca-1^+^/CD45^-^/CD34^+^ cell MCP-1 mRNA expression and MCP-1 secretion

To test effects of leptin *in vivo*, endotoxin-tested recombinant mouse leptin was injected into *ob/ob* mice. Leptin replacement increased epididymal adipose tissue MCP-1 mRNA levels (Figure 3A, *p*<0.001) and plasma MCP-1 (Figure 3B, *p*<0.001).

### Osteogenic, chondrogenic, and adipogenic differentiation and cell surface immunophenotypes of Sca-1^+^/CD45^-^/CD34^+^ cells

To demonstrate that Sca-1^+^/CD45^-^/CD34^+^ cells from mouse adipose tissue were MSC, their ability to differentiate into 3 major mesodermal lineages, i.e., osteoblasts, chondrocytes, and adipocytes was tested. Upon induction with optimized osteogenic differentiation cocktail, the cell monolayer was extensively covered by Alizarin red-positive calcified extracellular matrix (Additional file 1: Figure 1A, osteogenic differentiation cocktail (+)) whereas untreated cells did not show morphological change and remained negative for Alizarin red staining (Additional file 1: Figure 1A, osteogenic differentiation cocktail (−)). Osteo-induced cells expressed mRNAs for osteogenic markers, i.e. *Runx2*, *Col1a*, *Osterix*, *Bsp*, and *Osteocalcin,* all of which were undetectable after 30 cycles of PCR in control cells (not treated with induction cocktail) (Additional file 1: Figure 1A). Sca-1^+^/CD45^-^/CD34^+^ cells were seeded at high density in micromass culture and treated with chondrogenic induction cocktail that induced the formation of three-dimensional chondrogenic cell aggregates within 24 hours (Additional file 1: Figure 1B, chondrogenic differentiation cocktail (+)), whereas untreated cells remained as high-density monolayer cultures (Additional file 1: Figure 1B, chondrogenic differentiation cocktail (−)). Treated micromass stained positive for Alcian blue within 2 weeks, indicating the presence of sulfated proteoglycans, whereas untreated monolayer did not show any such staining (Additional file 1: Figure 1B, Alcian blue (+)). Cells in the induced micromass showed specific expression of *Col2α*, *Comp*, *Col10a*, and *Sox9* mRNAs associated with chondrogenesis, all of which were undetectable in monolayer of cells without induction cocktail (Additional file 1: Figure 1B). Following the induction with adipocyte differentiation cocktail, fat globules were noticed within 3–5 days (Additional file 1: Figure 1C, adipogenic differentiation cocktail (+)). These cells stained positive with Oil Red O while no differentiation was observed in the untreated controls (Additional file 1: Figure 1C). Expression of mRNAs for adipogenesis genes *Pparγ2*, *Cebpα*, *Fabp4,* and *Lpl* were expressed only in cells that were treated with adipogenic differentiation cocktail (Additional file 1: Figure 1C). We used the same cells to determine presence of stem cell surface markers stem cell antigen-1 (Sca-1) and for the absence of potential hematopoietic cell marker CD45. Data showed that these CD34^+^ cells were totally negative for CD45 (hematopoietic stem cell marker) and 95.5 ± 4.04 % of these cells were positive for Sca-1 (mesenchymal stem cell marker) (Additional file 1: Figure 1D-1F).

### Leptin induces MCP-1 secretion and MCP-1 mRNA expression

**Figure 4:**
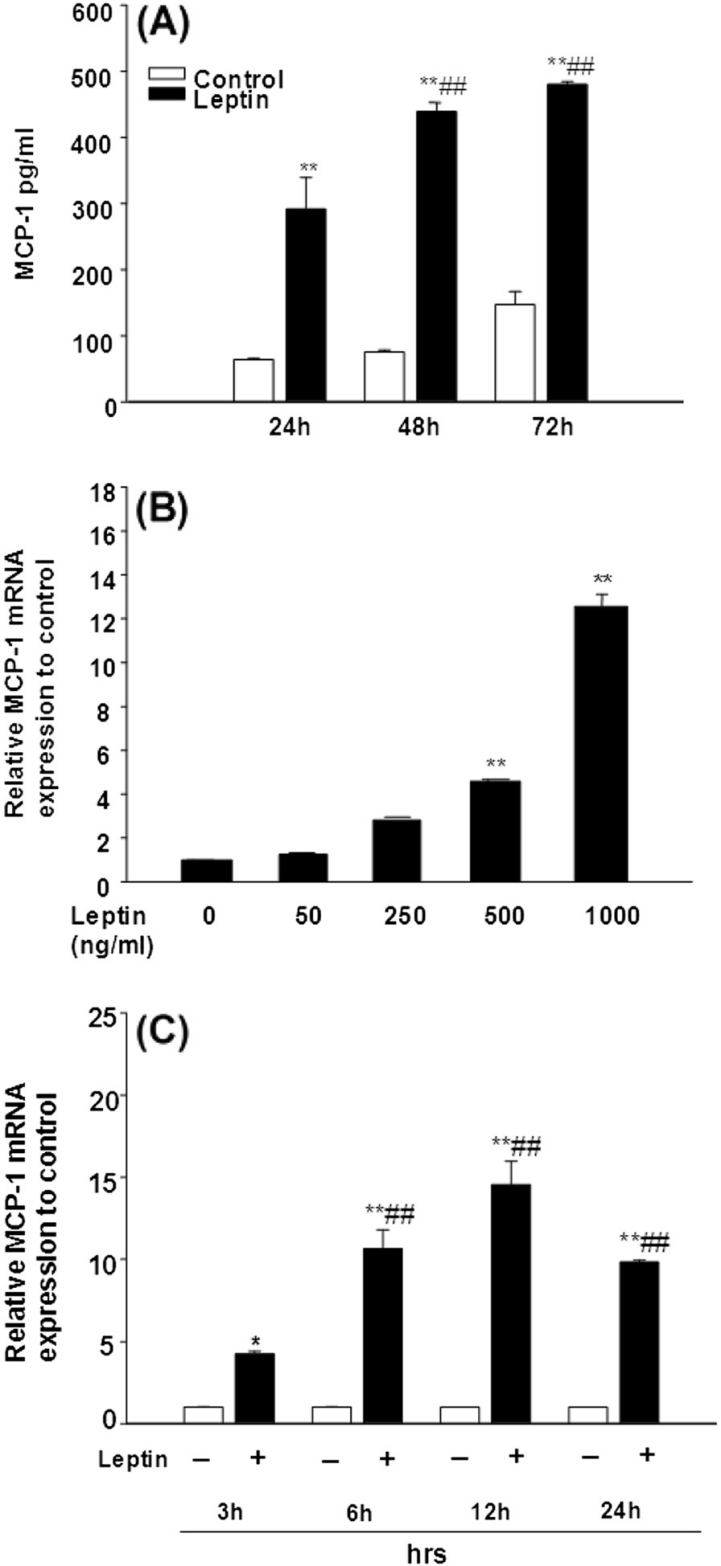
**Leptin induces MCP-1 secretion and mRNA expression in adipose stem cells in dose- and time-dependent manner.** (**A**). The *ob/ob* mice Sca-1^+^/CD45^-^/CD34^+^ cells seeded as 1 × 10^6^/ml/well in a 24-well culture plate and incubated with leptin (1000 ng/ml) for 24, 48, and 72 h. The supernatants were collected and MCP-1 protein levels were determined using ELISA. Data are expressed as mean ± SD, *n*=3. **p<0.01 compared to the value for untreated control cultures. ##*p*<0.01 compared to the value for treated with leptin 24 hours cultures. (**B**). The *ob/ob* mice Sca-1^+^/CD45^-^/CD34^+^ cells were seeded as 5×10^6^/5ml/well in a 6-well culture plate and incubated for 6 h with various doses of leptin as indicated. (**C**). The cells were seeded in a same density as in (B) with or without leptin (1000 ng/ml) for 3, 6, 12, and 24 h. For (B) and (C), total RNA were extracted and relative MCP-1 mRNA expression was determined by real time PCR and normalized against 18S-rRNA. Data are expressed as mean ± SD, n=3. For (B) and (C), *p<0.05, **p<0.01 compared to the value for untreated control cultures and ##*p*<0.01 compared to the value for cultures treated with leptin for 3 h cultures.

To determine whether leptin induces MCP-1 secretion, adipose CD34^+^ stem cells were treated with murine recombinant leptin (1000 ng/ml) for 24, 48 and 72 h. Leptin significantly and time-dependently increased MCP-1 secretion compared to control at all time points (Figure 4A, p<0.05). MCP-1 secretion at 48 and 72h further increased compared to 24h (Figure 4A, p<0.01) and reached a plateau between 48 h to 72 h. This suggests that MCP-1 secretion can be maximally induced by leptin as early as 48 h with the dose of 1000 ng/ml. To test whether leptin induces MCP-1 mRNA expression in adipose stem cells, we treated cells with varying doses of leptin (0, 50, 250, 500, and 1000 ng/ml) for 6 h and measured MCP-1 mRNA using real time RT-PCR. Leptin significantly increased MCP-1 mRNA levels when added at 500, and 1000 ng/ml (Figure 4B, *p*<0.01). Furthermore, leptin-induced MCP-1 mRNA expression in adipose Sca-1^+^/CD45^-^/CD34^+^ cells was significantly enhanced compared to control in a dose-dependent manner and this induction was time-dependent with peak expression observed at 12h of leptin stimulation (Figure 4C, *p*<0.01).

### Leptin–induced MCP-1 secretion is blocked by inhibition of JAK-2, PI3K, ERK, or Akt

**Figure 5:**
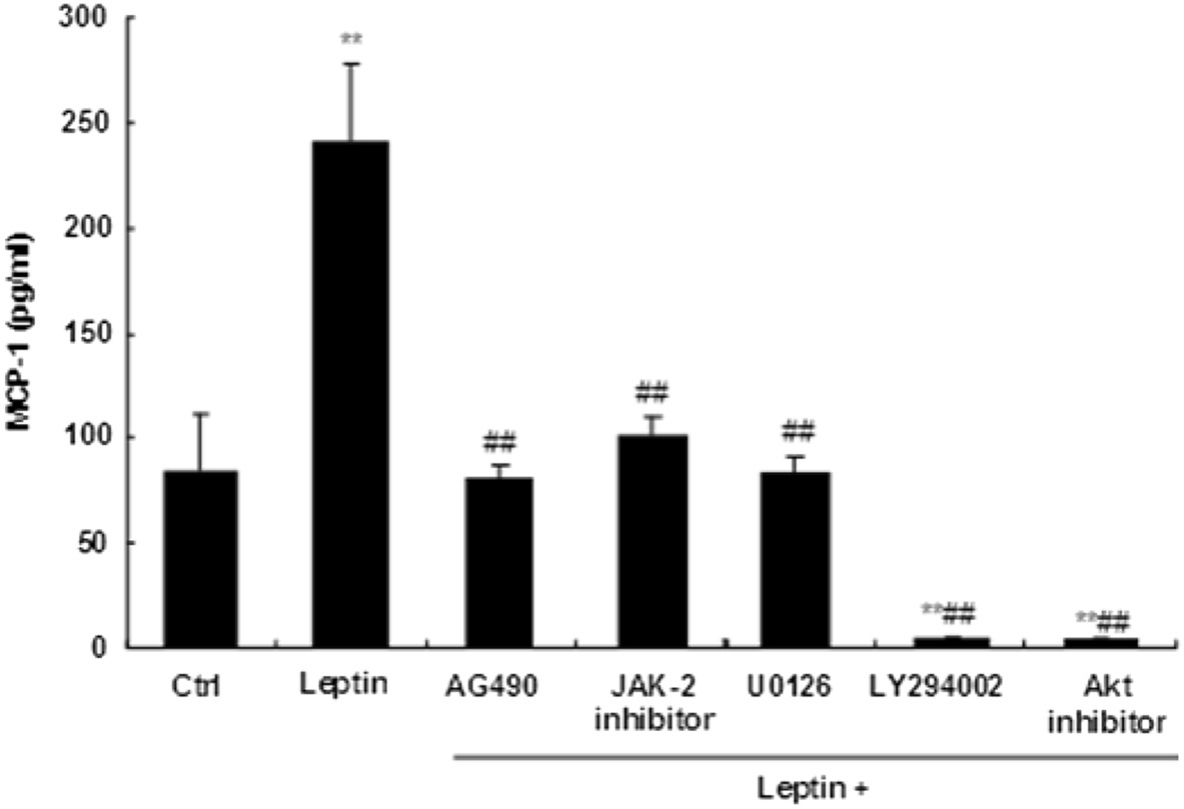
**Leptin induced MCP-1 secretion is decreased by JAK-2, ERK, PI3K, and Akt inhibitors.** The Sca-1^+^/CD45^-^/CD34^+^ cells from ob/ob mice were seeded as 1 × 10^6^/ml/well in a 24-well culture plate and pretreated with or without 50 μM of the JAK 2 inhibitor AG490, 0.5 μM of the JAK2 inhibitor JAK inhibitor I, 10 μM of the ERK inhibitor U-0126, 200 μM of the PI3 Kinase inhibitor LY294002, and 10 μM of the Akt inhibitor Akt inhibitor IV for 1 h. After 1h pretreatment, cell culture media were removed and cells were treated with leptin (1000 ng/ml) in media containing the same concentration of inhibitor for additional 24h. The supernatants were collected and determined by MCP-1 ELISA. Data are expressed as mean ± SD. **p<0.01 compared to the value for untreated control cultures. ##*p*<0.01 compared to the value for cultures subjected to leptin without inhibitor pretreatment.

To determine whether JAK2 and PI3K/Akt signal pathways participate in MCP-1 secretion induced by leptin, Sca-1^+^/CD45^-^/CD34^+^ cells were pretreated with or without the JAK-2 inhibitor (AG490), ERK inhibitor (U-0126), PI3 Kinase inhibitor (LY294002) for 1 hour. Cells were then treated with 1000 ng/ml leptin for additional 24 hours. All protein inhibitors that were tested suppressed the leptin-induced MCP-1 production (Figure 5, *p*<0.01). Cell viability (MTT assays) results suggest that reduction in MCP-1 secretion is not due to cell toxicity (data not shown).

### Leptin-dose dependently activates STAT3, PI3K, Akt, and ERK1/2

**Figure 6:**
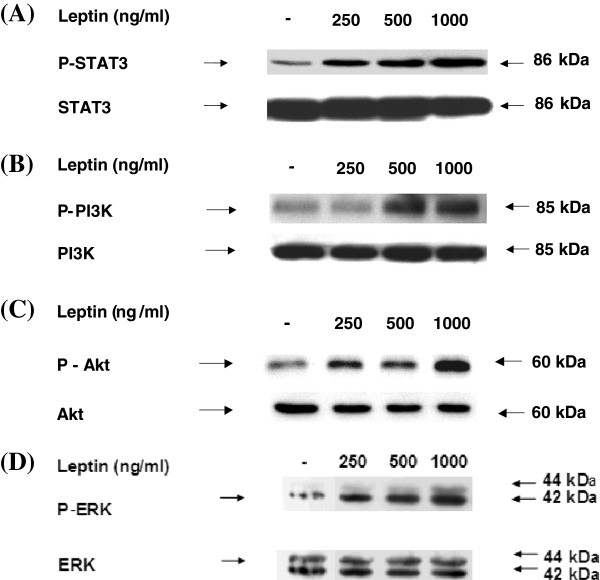
**Leptin-dose dependently increases activation of STAT3, PI3K, Akt, and ERK1/2.** The Sca-1^+^/CD45^-^/CD34^+^ cells from ob/ob mice were seeded as 1 × 10^7^/10ml/dish in a 100 mm × 20 mm culture dish. The cells were incubated with various dose of leptin (0, 250, 500 and 1000 ng/ml) for 30 min. Total cellular proteins were extracted and subjected to western blot analysis with antibodies specific for (**A**). anti-phospho-STAT3, (**B**). anti-phospho-PI3K p85(Tyr458), (**C**). anti-phospho-Akt, and (**D**). anti-phospho-p44/42 ERK (Thr202/Tyr204) as indicated. Bands were detected using the ECL system. Afterward, blots were stripped and reprobed with anti-STAT3, anti-PI3K p85, anti-Akt (pan), and anti-p44/42 ERK antibody for assessment of protein loading. Results shown are representative of three independent experiments.

To determine whether leptin activates JAK-STAT and downstream signaling regulators, Sca-1^+^/CD45^-^/CD34^+^ cells were treated with leptin concentrations of 0, 250, 500 to 1000 ng/ml to test whether phosphorylation of these leptin signaling intermediates were altered. Leptin increased phosphorylation of STAT3, Akt, and ERK1/2 at dose ranges of 250 to 1000 ng/ml while increased phosphorylation of PI3K was observed at 500 to 1000 ng/ml leptin (Figure 6).

### Leptin-mediated STAT3, PI3K, Akt, and ERK1/2 activation is prevented by JAK-2 inhibitor

**Figure 7:**
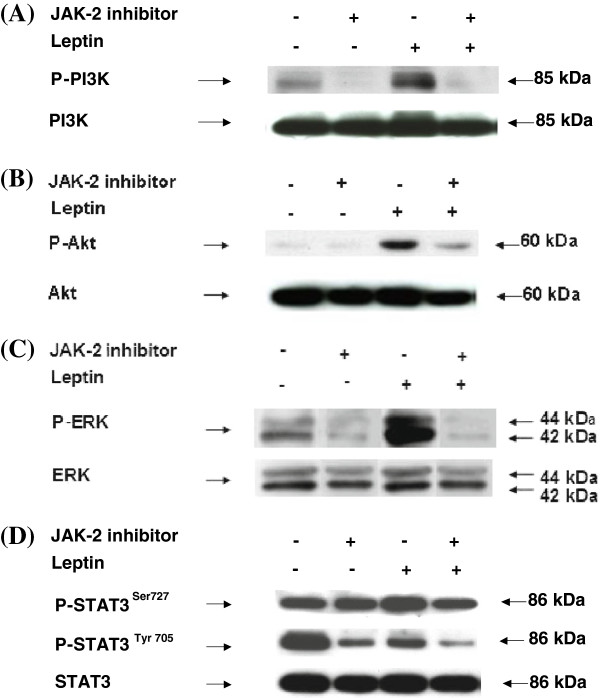
**Leptin–induced MCP-1 expression is inhibited by JAK-2 inhibitor, PI3K inhibitor, ERK inhibitor and Akt inhibitor.** The Sca-1^+^/CD45^-^/CD34^+^ cells were seeded as 1 ×10^7^/10ml/dish in a 100 mm × 20 mm culture dish. The cells were pre-incubated with 50 μM of the JAK 2 inhibitor AG490 for 1 h. After 1 hr, cell culture media was removed and fresh media containing leptin (1000 ng/ml) and 50 μM of the JAK2 inhibitor was added for additional 30 minutes. Total cellular proteins were extracted and subjected to western blot analysis with (**A**). anti-phospho-PI3K, (**B**). anti-phospho-Akt (**C**). anti-phospho-p44/42 ERK, and (**D**), anti-phospho-STAT3 (Ser727) and anti-phospho-STAT3 (Tyr705) antibody as indicated. Bands were detected using the ECL system. Afterward, blots were stripped and reprobed with anti-STAT3, anti-PI3K p85, anti-Akt (pan), and anti-p44/42 ERK antibody for assessment of protein loading. Results shown are representative of three independent experiments.

To test if inhibition of JAK-2 results in decreases in downstream leptin signaling pathway, Sca-1^+^/CD45^-^/CD34^+^ cells were treated with JAK-2 inhibitor and tested for phosphorylation of PI3K, Akt, ERK1/2 and STAT3. Leptin-induced phosphorylation of PI3K, Akt, ERK1/2 and STAT3 were inhibited by JAK-2 inhibitor (Figure 7A- 7D).

### Determination of downstream leptin signaling pathway sequence

**Figure 8:**
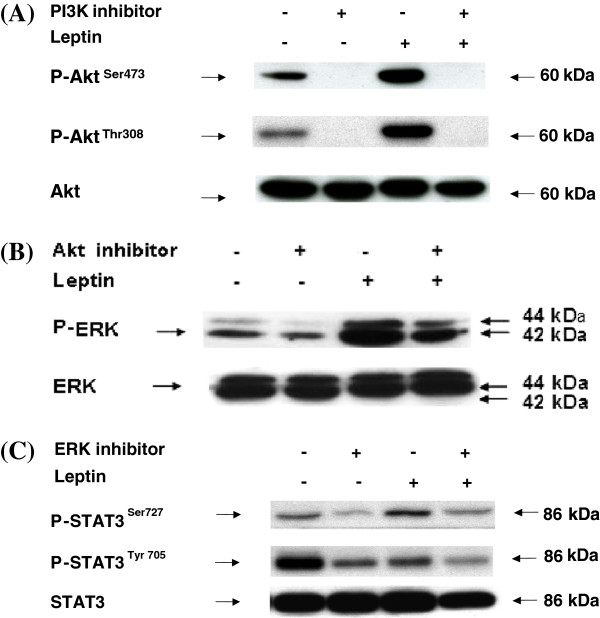
**Determination of downstream leptin signaling pathway sequence.** The Sca-1^+^/CD45^-^/CD34^+^ cells were seeded as 1 × 10^7^/10ml/dish. Cells were then pre-incubated with 200 μM of the PI3 Kinase inhibitor LY294002, 10 μM of the Akt inhibitor IV, and 10 μM of the ERK inhibitor U-0126 for 1 h. After 1 hr, cell culture media was removed and fresh media containing leptin (1000 ng/ml) and the same inhibitors were added for additional 30 min. Total cellular proteins were extracted and subjected to western blot analysis with (**A**). anti-phospho-Akt (**B**). anti-phospho-p44/42 ERK, and (**C**), anti-phospho-STAT3 (Ser727) and anti-phospho-STAT3 (Tyr705) antibody as indicated. Bands were detected using the ECL system. Afterward, blots were stripped and reprobed with anti-STAT3, anti-PI3K p85, anti-Akt (pan), and anti-p44/42 ERK antibody for assessment of protein loading. Results shown are representative of three independent experiments.

To determine the sequence of events that leads to leptin-induced MCP-1 gene expression in Sca-1^+^/CD45^-^/CD34^+^ cells, cells were treated with leptin. Starting with a well-established leptin JAK-STAT signaling pathway, PI3K inhibitor was added to determine if phosphorylation of expected downstream activator-Akt could be inhibited. PI3K inhibitor decreased expression of phosphorylated Akt (Figure 8A). We then tested if Akt inhibitor decreased leptin-induced Akt and ERK1/2 phosphorylation. Inhibition of Akt phosphorylation prevented ERK1/2 phosphorylation (Figure 8B). In addition, ERK inhibitor-mediated decreases in phosphorylated STAT3 reduced leptin-induced STAT3 phosphorylation at Ser 727 (Figure 8C). High levels of phosphorylated STAT3 Tyr 705 were observed under basal conditions and this unexpected high expression of phosphorylated STAT3 Tyr 705 was decreased in the presence of ERK inhibitor and leptin.

**Figure 9:**
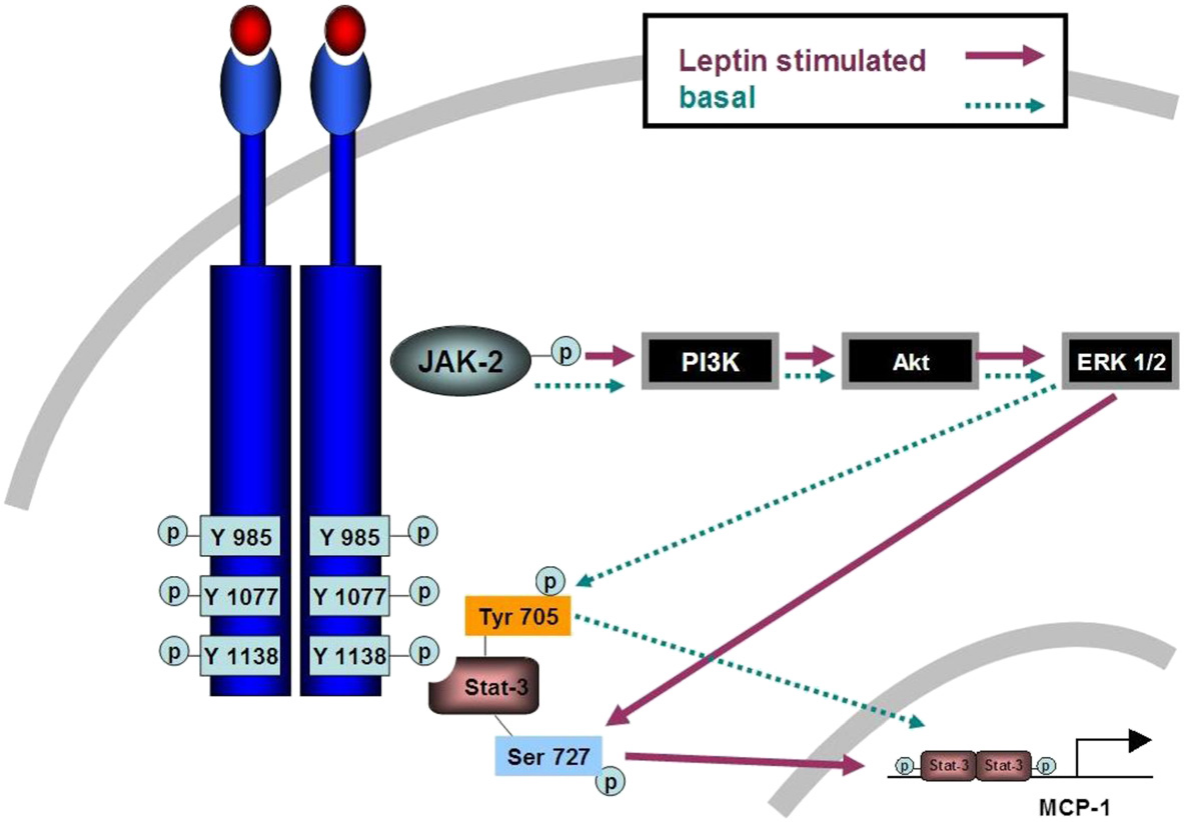
**Proposed leptin signaling pathway in adipose Sca-1**^**+**^**/CD45**^**-**^**/CD34**^**+**^**MSC cells.** Post leptin-leptin receptor binding and receptor-receptor dimerization, JAK-2 is phosphorylated. Activation of JAK-2 results in phosphorylation of PI3K which lead to phosphorylation of Akt and ERK1/2. Leptin-induced increase in phosphorylated ERK1/2 induces activation of STAT3 by phosphorylating Ser 727 amino acid residue. Under basal condition, alternative STAT3 Tyr 705 gets phosphorylated. Consequently, activated STAT3 translocates to nucleus and induces MCP-1 gene transcription in Sca-1^+^/CD45^-^/CD34^+^ MSC cells.

## Discussion

The current study demonstrated that *ob/ob* mice exhibit significantly greater adipose tissue mass, SVF and adipose MSC cell numbers as well as greater circulating MCP-1 and adipose MCP-1 expression. Data also showed that mesenchymal stem cells from adipose tissue of adult *ob/ob* mice exhibit hyperplasia and are responsive to obesity-related endocrine factors such as leptin. Noteworthy is that leptin deficient mice exhibit hyperplasia partly due to increased concentrations of other obesity-associated endocrine and mitogenic factors such as insulin and IL-6. Although an absence of leptin in ob/ob mice still showed increased inflammation compared to the lean mice, data from our current study showed that injected leptin cause even greater hyperplasia beyond the leptin-deficient state. Data demonstrating decreases in SVF cell numbers per gram of adipose tissue in *ob/ob* mice and decreases in percent of CD34^+^/Sca-1^+^/CD45^-^ cells in SVF in *ob/ob* mice were opposite to our initial expectation that obesity induces hyperplasia of adipocytes as well as their precursor cells including SVF and CD34^+^/Sca-1^+^/CD45^-^ cells. However, our data are consistent with another study which showed an increase in the total number of stromal cells with increased adiposity and a negative correlation between adiposity and number of stromal cells per gram of adipose tissue [39].

Added statement (line 3, p 17): In our current study we showed that plasma levels of MCP-1 in ob/ob mice, is elevated compared to control mice, in spite of leptin deficiency. However, it is plausible that ob/ob mice have elevated MCP-1 because of obesity that is a secondary response to leptin deficiency. To address whether leptin can directly upregulate MCP-1, we gave ob/ob mice a single injection of leptin for short term (3 hrs) and also long term (7 days). The plasma MCP-1 levels measured from ob/ob mice injected with leptin for 7 days showed significant decreases in body weight as demonstrated previously [40] and at the same time plasma MCP-1 levels decreased significantly (55 + 4.5 pg/ml) compared to ob/ob mice that were injected with saline for 7 days as a control (67 + 4.7 pg/ml). Taken together these results suggest that that leptin itself can directly upregulate MCP-1 and that MCP-1 levels in ob/ob mice are decreased subsequent to body weight reduction.

We previously reported that the basal adipose tissue MCP-1 mRNA level was greater in the leptin deficient *ob/ob* mice, possibly due to a greater number of MCP-1 secreting adipose stem cells [5]. The current study showed that leptin-treated *ob/ob* mice exhibit greater levels of adipose tissue MCP-1 mRNA and plasma MCP-1. Using adipose MSC cells isolated from *ob/ob* mice, we further demonstrated that the number of these adipose MSC cells is increased proportionally to adipose tissue mass. Moreover, we determined that these cells exhibit MSC phenotypes that include multilineage differentiation potential into mesenchymal cell types and showed that these cells express well-known markers of MSC (Additional file 1: Figure 1). One noteworthy finding of this study is that cell surface CD45 antigen is absent in these adipose MSC cells.

To date, no studies have addressed whether obesity-associated endocrine factors such as leptin, regulate adipose stem cell-derived MCP-1, nor have they dissected the intracellular signaling intermediates involved in leptin-mediated MCP-1 gene expression in the stem cells. Accordingly, we sought to characterize the molecular mechanisms underlying obesity-associated increases in adipose tissue MCP-1 concentrations. Specifically, we characterized leptin-induced intracellular signaling pathways that lead to increased MCP-1 secretion and gene expression. We identified two separate signaling pathways leading to MCP-1 gene expression regulation in Sca-1^+^/CD45^-^/CD34^+^ adipose MSC cells. Under the basal conditions, these cells express constitutively activated Tyr705 in STAT3, which was decreased by leptin treatment. In contrast, STAT3 Ser 727 was expressed at low levels in basal conditions and was activated by leptin. The exact mechanism of this differential regulation of STAT3 Tyr705 and Ser727 is not known. Activation of the STAT3 first requires STAT3 Tyr705 phosphorylation-induced dimerization of the cytokine receptor and the STAT3 activity is subsequently modulated by phosphorylation at Ser727 with various stimuli [41,42]. Other studies have suggested that activation of STAT Ser727 is independent of activation of STAT3 Tyr705 [42] and that while both Tyr705 and Ser 727 are essential for full activation of STAT3 pathway, only STAT3 Ser 727 phosphorylation is triggered by exogenous stimuli such as insulin, TNF-alpha, and lipopolysaccharide [42]. In addition, exposure of mouse epidermal tumor prone JB6 cells to UV radiation triggered only STAT3 Ser 727 phosphorylation and not STAT3 Tyr705 [42]. Downstream kinase of ERK that can independently activate Ser 727 and Tyr705A of STAT3 has been explored and the findings suggested that mitogen- and stress-activated protein kinase 1 (MSK1) may specifically phosphorylate STAT3 Ser 727 [42]. Interestingly, STAT3 Ser727 phosphorylation negatively regulates STAT3 Tyr705 by dephosphorylating STAT3 Tyr705 [43]. In one study, leptin added at 2 nM stimulated phosphorylation of both STAT3 Tyr705 and Ser727 in murine macrophage cell line J774.2 cells [41]. This finding contrasts our current study in which leptin decreased constitutively and highly expressed phopho-STAT3 Tyr705, only if added at concentrations higher than 50 nM. This difference may be related to the difference in cell types used in two studies, i.e., transformed J774.2 cells vs. primary Sca-1^+^/CD45^-^/CD34^+^ cells in our study. It is plausible that observed high basal levels of STAT3 Tys705 in our *ob/ob* mice adipose MSC cells may be indicative of malignant phenotype or transformed cells. However, our data from the anchorage-independent growth (AIG) test (used to test for malignancy by characterizing the cells that do not require a solid platform such as the plastic surface of the culture dish for their growth) demonstrated that our Sca-1^+^/CD45^-^/CD34^+^ cells are not cancerous cells (data not shown). The possible reason for very high levels of phospho-STAT3 Tyr 705 is currently unknown and warrants further investigation.

## Conclusions

Our data demonstrated that adipose MSC cell number significantly increased with leptin injection and that leptin up-regulated MCP-1 secretion in a dose- and time-dependent manner. We further showed that increases in MCP-1 mRNA levels in the adipose stem cells were due to increased phosphorylation of STAT3 Ser727, but not due to changes in STAT3 Tyr705 phosphorylation suggesting differential regulation of MCP-1 gene expression under basal and leptin-stimulated conditions in adipose stem cells (Figure 9). Identification of factors regulating adipose MSC cell number and production of proinflammatory adipokine such as MCP-1, as well as dissection of leptin modulated signaling pathway in these cells may help in developing novel anti-obesity target strategies.

## Abbreviations

Ad-MSC: Adipose-derived mesenchymal stem cell; AIG: Anchorage-independent growth; AG: β-glycerophosphate disodium; Asc: 2P L-ascorbic acid 2-phosphate; CVD: Cardiovascular Disease; DAG: β-glycerophosphate disodium; FAC: Fluorescence activated cell sorting; IL: Interleukin; JAK: Janus kinase; Μϕ: Macrophag; MCP-1: Monocyte chemotactic protein-1; MSC: Mesenchymal stem cell; Ob-R: Leptin receptor; PE: R-phycoerythrin; Sca-1: Stem cell antigen 1; STAT: Signal transducer and activator of transcription; SVF: Stromal vascular fraction; T2D: Type 2 diabetes; TGF-β1: Transforming growth factor-beta 1; TNF-α: Tumor necrosis factor-alpha.

## Competing interests

Authors have no competing conflict of interests.

## Authors’ contributions

ZZ, MN, HRZ, CCC, NMM, and KC designed research; ZZ, MN, HRZ, CCC, DW, and KC performed research; ZZ, MN, HRZ, CCC, DW, NMM and KC analyzed data; and ZZ, MN, HRZ, CCC, DW, NMM and KC wrote the paper. All authors read and approved the final manuscript.

## Supplementary Material

Additional file 1 **Figure 1.** Sca-1^+^/CD45^-^/CD34^+^ cells from *ob/ob* mice have multilineage differentiation potential. (**A**). Osteogenic differentiation of mouse adipose tissue derived CD34^+^ mAd-MSCs. MACS-separated cells were treated with osteogenic differentiation cocktail for 4 weeks. Alizarin red-positive mineralized deposits were present in the cells that were treated with osteogenic differentiation cocktail (+), but not in control cells that were not treated with osteogenic differentiation cocktail (−). Cells were treated with and without osteogenic differentiation cocktail for total RNA isolation. Levels of mRNA for osteogenic marker gene *Runx2, Col1a1*, *Osterix* (*Osx*), *Osteocalcin (Oc), Bone sialoprotein* (*Bsp*) and housekeeping *Gapdh* gene were determined using RT-PCR methods. (**B**). Chondrogenic differentiation of mouse adipose tissue derived CD34^+^ mAd-MSCs. Cells were treated with chondrogenic differentiation cocktail for 2 weeks. Formation of micromass that is stained positive with sulfated proteoglycan-specific Alcian blue was seen in cells that were treated with chondrogenic differentiation cocktail (+) but not in control cells that were not treated with chondrogenic differentiation cocktail (−). Cells were treated with and without chondrogenic differentiation cocktail for total RNA isolation. Levels of mRNA for chondrogenic marker gene *Col2a*, *Col10a*, *Comp*, *Sox9* and housekeeping *Gapdh* gene were determined using RT-PCR methods. (**C**). Adipogenic differentiation of mouse adipose tissue derived CD34^+^ mAd-MSCs. Cells were treated with adipogenic differentiation cocktail for 2 weeks. Lipid accumulation was detected using Oil Red O staining in cells that were treated with adipogenic differentiation cocktail (+) but not in control cells that were not treated with adipogenic differentiation cocktail (−). Cells were treated with and without adipogenic differentiation cocktail for total RNA isolation. Levels of mRNA for adipogenic marker gene *Pparγ2*, *Cebpα*, *Fabp4*, *Lpl* and housekeeping *Gapdh* gene were determined using RT-PCR methods. For (A)-(C), images (100×) are representative of three independent experiments and RT-PCR results are from pooled total RNA sample of three independent samples. Click here for file
