# Supplementary figures and images for: Leptin differentially regulate STAT3 activation in ob/ob mouse adipose mesenchymal stem cells
Source: Nutr Metab (Lond). 2012 Dec 5;9:109. doi: 10.1186/1743-7075-9-109 (PMC3559810; doi:10.1186/1743-7075-9-109)

**Additional Figure 1.**

**
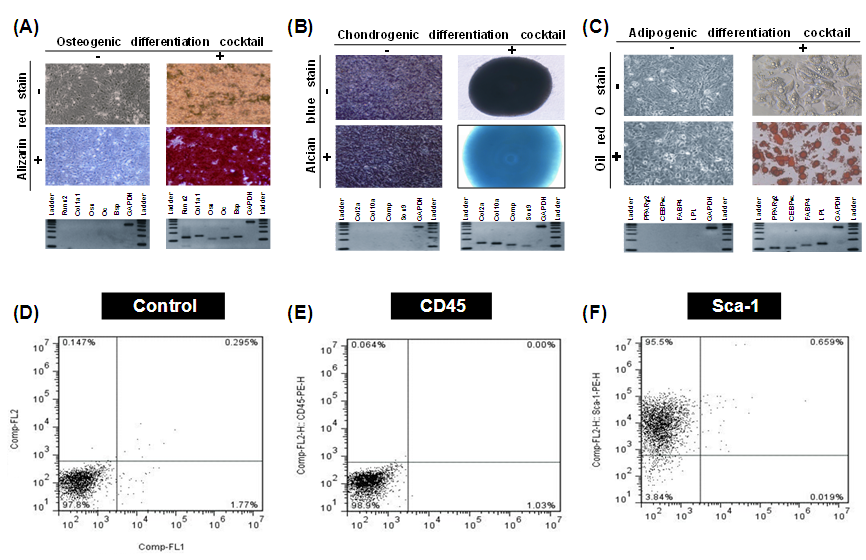
**

Supplement: Additional file 1 — Figure 1. Sca-1+/CD45-/CD34+ cells from ob/ob mice have multilineage differentiation potential. (A). Osteogenic differentiation of mouse adipose tissue derived CD34+ mAd-MSCs. MACS-separated cells were treated with osteogenic differentiation cocktail for 4 weeks. Alizarin red-positive mineralized deposits were present in the cells that were treated with osteogenic differentiation cocktail (+), but not in control cells that were not treated with osteogenic differentiation cocktail (−). Cells were treated with and without osteogenic differentiation cocktail for total RNA isolation. Levels of mRNA for osteogenic marker gene Runx2, Col1a1, Osterix (Osx), Osteocalcin (Oc), Bone sialoprotein (Bsp) and housekeeping Gapdh gene were determined using RT-PCR methods. (B). Chondrogenic differentiation of mouse adipose tissue derived CD34+ mAd-MSCs. Cells were treated with chondrogenic differentiation cocktail for 2 weeks. Formation of micromass that is stained positive with sulfated proteoglycan-specific Alcian blue was seen in cells that were treated with chondrogenic differentiation cocktail (+) but not in control cells that were not treated with chondrogenic differentiation cocktail (−). Cells were treated with and without chondrogenic differentiation cocktail for total RNA isolation. Levels of mRNA for chondrogenic marker gene Col2a, Col10a, Comp, Sox9 and housekeeping Gapdh gene were determined using RT-PCR methods. (C). Adipogenic differentiation of mouse adipose tissue derived CD34+ mAd-MSCs. Cells were treated with adipogenic differentiation cocktail for 2 weeks. Lipid accumulation was detected using Oil Red O staining in cells that were treated with adipogenic differentiation cocktail (+) but not in control cells that were not treated with adipogenic differentiation cocktail (−). Cells were treated with and without adipogenic differentiation cocktail for total RNA isolation. Levels of mRNA for adipogenic marker gene Pparγ2, Cebpα, Fabp4, Lpl and housekeeping Gapdh gene [file 1743-7075-9-109-S1.doc]
